# Subclinical Myocardial Fibrosis in South African Youth With HIV: Results From the CTAAC-Heart Study

**DOI:** 10.1093/ofid/ofae555

**Published:** 2024-10-01

**Authors:** Jennifer Jao, Heather J Zar, Morné Kahts, Stephen Jermy, Daniel Egan, Mothabisi N Nyathi, Nana Akua Asafu-Agyei, Justine Legbedze, Emma Carkeek, Nomawethu Jele, Tafadzwa Mautsa, Lauren Balmert Bonner, Grace A McComsey, Matthew Feinstein, Irwin J Kurland, Landon Myer, Ntobeko A B Ntusi

**Affiliations:** Division of Pediatric Infectious Diseases, Division of Adult Infectious Diseases, Department of Pediatrics, Department of Medicine, Feinberg School of Medicine, Northwestern University, Chicago, Illinois, USA; Department of Pediatrics and Child Health and SA-MRC Unit on Child and Adolescent Health, University of Cape Town, Cape Town, South Africa; Division of Cardiology, Department of Medicine, University of Cape Town, Cape Town, South Africa; University of Cape Town/South African Medical Research Council Extramural Unit on Intersection of Noncommunicable Diseases With Infectious Diseases; University of Cape Town/South African Medical Research Council Extramural Unit on Intersection of Noncommunicable Diseases With Infectious Diseases; Division of Biomedical Engineering, Department of Human Biology, University of Cape Town, Cape Town, South Africa; Division of Cardiology, Department of Medicine, University of Cape Town, Cape Town, South Africa; University of Cape Town/South African Medical Research Council Extramural Unit on Intersection of Noncommunicable Diseases With Infectious Diseases; Division of Epidemiology and Biostatistics, School of Public Health, University of Cape Town, Cape Town, South Africa; Department of Pediatrics and Child Health and SA-MRC Unit on Child and Adolescent Health, University of Cape Town, Cape Town, South Africa; Division of Pediatric Infectious Diseases, Department of Pediatrics, Ann and Robert H. Lurie Children's Hospital of Chicago, Chicago, Illinois, USA; Department of Pediatrics and Child Health and SA-MRC Unit on Child and Adolescent Health, University of Cape Town, Cape Town, South Africa; Department of Pediatrics and Child Health and SA-MRC Unit on Child and Adolescent Health, University of Cape Town, Cape Town, South Africa; Department of Pediatrics and Child Health and SA-MRC Unit on Child and Adolescent Health, University of Cape Town, Cape Town, South Africa; Department of Preventive Medicine, Feinberg School of Medicine, Northwestern University, Chicago, Illinois, USA; Department of Medicine, Department of Pediatrics, Case Western Reserve University, Cleveland, Ohio, USA; Department of Preventive Medicine, Feinberg School of Medicine, Northwestern University, Chicago, Illinois, USA; Division of Adult Cardiology, Department of Medicine, Feinberg School of Medicine, Northwestern University, Chicago, Illinois, USA; Division of Adult Endocrinology, Department of Medicine, Albert Einstein College of Medicine, Bronx, New York, USA; Division of Epidemiology and Biostatistics, School of Public Health, University of Cape Town, Cape Town, South Africa; Division of Cardiology, Department of Medicine, University of Cape Town, Cape Town, South Africa; University of Cape Town/South African Medical Research Council Extramural Unit on Intersection of Noncommunicable Diseases With Infectious Diseases; African Research Universities Alliance/The Guild of European Research-Intensive Universities Cluster of Research Excellence on Noncommunicable Diseases and Associated Multimorbidity

**Keywords:** antiretroviral, myocardial fibrosis, myocardial magnetic resonance imaging, perinatally acquired HIV, youth with HIV

## Abstract

**Background:**

Few data exist on myocardial fibrosis and inflammation in youth with HIV.

**Methods:**

We performed cardiovascular magnetic resonance (CMR) on a cross section of South African youth: youth with perinatally acquired HIV (YPHIV) undergoing antiretroviral therapy (ART), youth with nonperinatally acquired HIV (YNPHIV) receiving ART, and youth without HIV. Quantile regression models were fit to assess the association between HIV status and CMR outcomes: subclinical fibrosis (late gadolinium enhancement [LGE] mass and fraction, native T1, extracellular volume) and inflammation (native T1, T2 mapping).

**Results:**

Of 464 youth, 287 were YPHIV, 87 were YNPHIV, and 90 were HIV seronegative. The median age was 16 years (range, 11–24). LGE mass was higher in YPHIV and YNPHIV than in youth who were HIV seronegative (1.85 vs 2.00 vs 1.41 g, respectively), as was fraction (5.8% vs 6.4% vs 4.5%); native T1 was highest in YNPHIV. In adjusted analyses, when compared with youth with HIV seronegativity, YPHIV and YNPHIV exhibited higher LGE mass (β = 0.468, *P* = .001; β = 0.544, *P* = .002) and LGE fraction (β = 1.587, *P* < .001; β = 1.781, *P* < .001). CMR outcomes were similar between YPHIV and YNPHIV.

**Conclusions:**

Despite ART use, YPHIV and YNPHIV appear to have higher subclinical myocardial fibrosis than youth who are HIV seronegative and healthy adults in South Africa and may benefit from early screening/monitoring for cardiovascular disease.

## Teaser

Despite an absence of significant cardiovascular comorbidity, youth with perinatally or nonperinatally acquired HIV appear to have a higher degree of subclinical myocardial fibrosis than youth who are HIV seronegative.

## Graphical Abstract

**Graphical Abstract:**
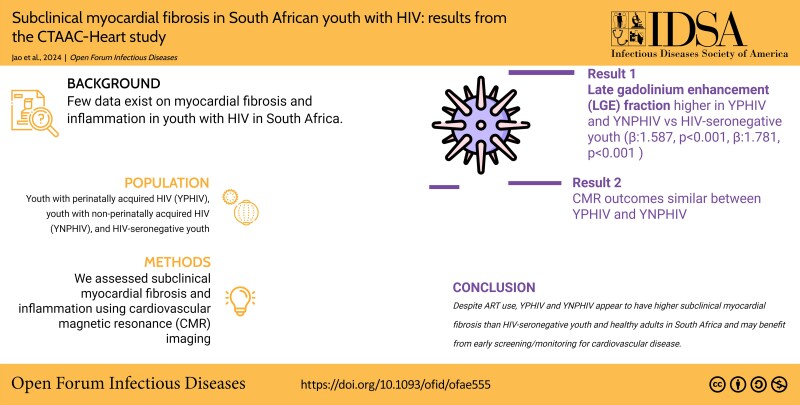
This graphical abstract is also available at Tidbit: https://tidbitapp.io/tidbits/subclinical-myocardial-fibrosis-in-south-african-youth-with-hiv-results-from-the-ctaac-heart-study?utm_campaign=tidbitlinkshare&utm_source=ITP

Increasing risk of cardiovascular disease (CVD) in adult persons with HIV (PWH) has become a major public health concern, and the World Health Organization now recommends that assessment and management of CVD risk be provided to all individuals with HIV [1]. Mounting evidence in adults shows that HIV, even when controlled with antiretroviral therapy (ART), is associated with elevated risks for myocardial dysfunction, heart failure, atherosclerotic CVD (including cerebrovascular and ischemic heart disease), sudden cardiac death, and CVD hospitalizations, as well as up to a 1.5-times higher risk for acute coronary syndrome as compared with the general population [2–5]. In addition, adult PWH exhibit greater myocardial fibrosis, inflammation, and steatosis in studies based on cardiovascular magnetic resonance (CMR) [6–15].

Despite the large body of literature surrounding cardiometabolic morbidity in adult PWH, similar studies in youth with HIV are lacking, particularly in sub-Saharan Africa, where 90% of the world's pediatric HIV population resides [16]. Little is known about subclinical or early upstream CVD pathogenesis in youth with perinatally acquired HIV (YPHIV) or youth with nonperinatally acquired HIV (YNPHIV), who stand to benefit the most from early identification and treatment of subclinical CVD. For example, youth with HIV often have not aged to a degree where traditional risk factors (diabetes, hypertension, dyslipidemia, etc) have firmly set in; thus, evaluating this younger population would be very important for understanding the effects of HIV and its treatment, without the confounding effects from traditional known risk factors on CVD outcomes.

Some effects of HIV infection include systemic inflammation, and inflammation has been postulated to play a role in myocardial injury [10, 17]. Several studies have shown higher cardiometabolic risk, subclinical atherosclerosis, as well as endothelial and myocardial dysfunction in YPHIV, but few have evaluated myocardial tissue fibrosis, inflammation, and deformational abnormalities by detailed CMR in this population [18–21]. CMR is a magnetic resonance imaging technique currently accepted as the noninvasive gold standard for measurement of myocardial tissue characteristics, structure, mass, and function, providing a valuable method for comprehensive and detailed assessments of these myocardial domains [22]. The objective of our study was to assess whether YPHIV and YNPHIV have worse subclinical myocardial fibrosis, inflammation, and function as compared with youth in South Africa who are HIV seronegative.

## METHODS

### Study Population

The Cape Town Adolescent and Antiretroviral Cohort (CTAAC) is a previously described observational study evaluating the long-term health of YPHIV well established with ART as compared with a HIV-seronegative cohort of youth in South Africa [23]. CTAAC-Heart is a substudy that enrolled CTAAC participants as well as YNPHIV and youth who were HIV seronegative in Cape Town, who were 12 to 24 years of age from October 2020 to August 2022, to achieve the following comparison groups: YPHIV, YNPHIV, and youth with HIV-seronegative status. All YPHIV in CTAAC-Heart had received ART for ≥6 months and YNPHIV for ≥1 month prior to enrollment. Pregnant individuals and those with congenital heart disease or preexisting renal disease (glomerular filtration rate <30 mL/min) were excluded, as were those with known in utero HIV exposure among youth who were HIV seronegative.

### Patient Consent Statement

All participants provided written informed consent and/or assent as appropriate. This study was approved by the institutional review boards of the University of Cape Town, Northwestern University Feinberg School of Medicine, and Ann & Robert H. Lurie Children's Hospital of Chicago.

### Primary Outcome

Subclinical myocardial fibrosis, inflammation, and function were assessed by CMR. Late gadolinium enhancement (LGE) mass and volume fraction as well as extracellular volume (ECV) were measured to assess subclinical myocardial focal and diffuse fibrosis, respectively; native T1 and T2 mapping were performed to evaluate for chronic and acute inflammation; strain, strain rate, and global left ventricular ejection fraction were measured to assess myocardial function. CMR was performed with a single 3-T magnetic resonance system (Skyra; Siemens Healthcare). All CMR images were stored and analyzed offline in blinded fashion in a dedicated core laboratory, with 2 independent readers with a minimum of 2 years’ experience using Circle CVI42 (Circle Cardiovascular Imaging). Where there was discordance, independent adjudication was provided by a third reader with a minimum of 15 years’ CMR experience. LGE was performed via a diastolic-triggered, inversion-prepared, 2-dimensional spoiled gradient echo sequence with gadolinium-DTPA (dose, 0.1–0.2 mmol/kg) with corresponding images in the short- and long-axis views. Quantitative analysis of LGE was employed for the presence of global enhancement (total left ventricle volume; percentage of enhanced volume = enhanced volume/total volume) with manual selection of normal area appearing as maximally suppressed myocardium. The enhanced volume was automatically calculated as the area of the myocardium 2 SD higher than the mean signal of the normal area. The presence of LGE was defined as an LGE fraction >5%. ECV was calculated from pre- and postcontrast modified Look-Locker inversion recovery T1 mapping sequences performed in 3 parallel short-axis slice acquisitions: basal, mid, and apical [24, 25]. T2 mapping was performed by a T2-prepared fast low-angle shot sequence done in 3 analogous short-axis slice acquisitions. Indices of regional function and deformation were measured by strain and strain rate, as well as biventricular ejection fraction, volume, and mass. Volumetric cavity assessment was obtained by whole-heart coverage of gapless short-axis slices via a balanced steady-state free precession cine sequence. For strain imaging, feature tracking was performed on the short-axis cine slices and the 4-chamber, 2-chamber, and left ventricular outflow tract long-axis slices to assess global radial, circumferential, and longitudinal strain and strain rates [26].

### Primary Exposure of Interest

The primary exposure of interest was HIV status and mode of acquisition (perinatal vs nonperinatal). HIV status was confirmed through previously collected data in the parent CTAAC study (perinatal HIV acquisition), medical record review (nonperinatal HIV acquisition), and HIV testing (HIV seronegativity).

### Covariates

Clinical and sociodemographic variables were collected through a combination of record review and questionnaires. Anthropometric and blood pressure measurements were performed by trained research staff in standardized fashion using calibrated scales, stadiometers, measuring tapes, and sphygmomanometers. Fasting glucose and insulin for HOMA-IR calculation (homeostatic model assessment–insulin resistance) [27] and lipids were obtained at the same time as the CMR, following previously described methodology [28].

### Statistical Analysis

CMR outcomes were compared among YPHIV, YNPHIV, and youth who were HIV seronegative by Kruskal-Wallis tests. Due to the nonlinear nature of the outcome variables, quantile regression models were fit to assess the association of HIV status with each of the following CMR outcomes: LGE mass, LGE volume fraction, mean ECV, mean native T1 time (across the base, mid, and apex areas). Subgroup analyses were conducted among youth with HIV to further assess the association of mode of HIV acquisition with CMR outcomes. Age, socioeconomic status (assessed by type of running water access), sex, body surface area, and previous tuberculosis were considered confounders a priori from known literature [29–31] in models with the entire cohort, and HIV RNA level [32] was additionally considered a confounder a priori in subgroup analyses of youth with HIV. Other variables were regarded as potential confounders if they were associated with any of the CMR outcomes at *P* ≤ .10. Sensitivity analyses were performed restricting models to females only. Alpha levels <.05 were deemed significant. All statistical analyses were conducted in Stata version 18 (StataCorp).

## RESULTS

**Figure 1.**
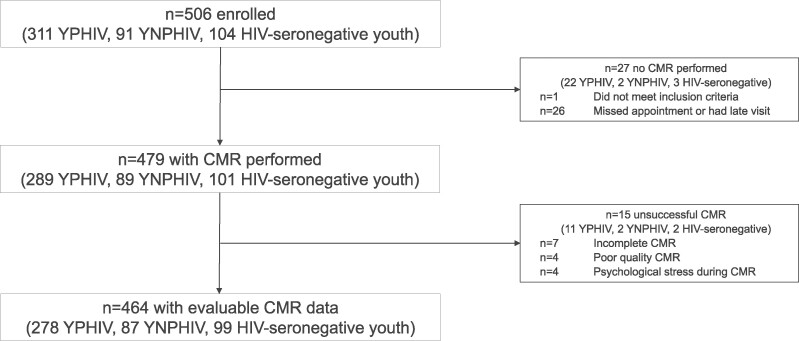
CTAAC-Heart study population derivation. CMR, cardiac magnetic resonance; YNPHIV, youth with nonperinatally acquired HIV; YPHIV, youth with perinatally acquired HIV.

A total of 464 youth were included in this analysis: 278 YPHIV, 87 YNPHIV, and 99 youth with HIV seronegativity (Figure 1). YNPHIV were older with a higher proportion self-identifying as female than YPHIV and youth who were HIV seronegative (median age 20 vs 18 and 17 years; 85% vs 50% and 49%, respectively; Table 1). YPHIV had a lower body surface area (1.6 vs 1.7 and 1.7 m^2^) and a higher proportion with prior tuberculosis disease (70% vs 20% and 8%) vs YNPHIV and youth with HIV seronegativity. Youth who were HIV seronegative had lower HOMA-IR (2.1 vs 2.6 and 2.4) and triglycerides (54.9 vs 65.5 and 62.8 mg/dL) when compared with YPHIV and YNPHIV. Smoking, total cholesterol, and low-density lipoproteins were similar among groups. Among youth with HIV, YPHIV had a higher proportion with CD4 <200 cells/mm^3^ (10% vs 4%) or viral load >50 copies/mL (39% vs 13%) but a lower proportion with integrase inhibitors (32% vs 84%).

**Table 1.** Characteristics of Participants by HIV Status

	YPHIV (n = 278)	YNPHIV (n = 87)	Youth Without HIV (n = 99)
Sociodemographic			
Age, y	18.4 (17.1–19.9)	20.8 (19.1–22.5)	17.2 (16.1–18.8)
Female	140 (50.4)	74 (85.1)	48 (48.5)
Home running water	244 (87.8)	62 (83.8)	51 (51.5)
Family history CVD	8 (3.0)	0 (0.0)	2 (2.0)
Smoking	33 (12.0)	16 (18.6)	10 (10.2)
Previous tuberculosis	195 (70.1)	17 (19.8)	6 (8.0)
Blood pressure, mm Hg			
Systolic	104.5 (100.3–112.3)	108.0 (103.0–117.7)	107.3 (101.7–114.7)
Diastolic	66.7 (62.0–71.8)	69.3 (64.7–76.0)	68.3 (63.0–74.3)
Tanner stage ≥4	210 (97.7)	80 (100.0)	68 (97.1)
Anthropometrics			
Body mass index, kg/m^2^	21.9 (19.6–25.2)	25.7 (21.4–30.3)	21.1 (19.2–26.3)
Body surface area, m^2^	1.6 (1.5–1.7)	1.7 (1.6–1.9)	1.7 (1.5–1.8)
Waist circumference, cm	73.0 (69.0–80.0)	81.0 (71.0–91.0)	73.0 (68.0–82.0)
Waist:height ratio	45.6 (42.1–50.8)	50.5 (44.4–56.0)	43.9 (41.5–51.7)
HOMA-IR	2.6 (1.7–4.2)	2.4 (1.6–3.9)	2.1 (1.1–3.7)
Fasting lipid, mg/dL			
Total cholesterol	139.4 (115.1–159.8)	124.3 (112.7–146.3)	129.3 (110.4–154.8)
LDL	74.9 (60.2–91.9)	66.0 (53.3–84.9)	70.0 (54.1–91.1)
HDL	46.3 (37.8–54.8)	44.4 (37.5–53.7)	45.6 (40.9–57.1)
Triglycerides	65.5 (51.3–86.7)	62.8 (46.0–82.3)	54.9 (45.1–67.3)
HIV-specific factors			
CD4, cells/mm^3^			
<200	28 (10.3)	3 (3.7)	…
201–500	92 (33.7)	29 (35.4)	…
>500	153 (56.0)	50 (61.0)	…
Log HIV RNA level	3.9 (3.7–6.7)	3.7 (3.7–3.9)	…
Age at ART initiation, y	3.8 (1.8–6.7)	19.0 (16.9–20.7)	…
Duration, y			
HIV infection	18.45 (17.14–19.94)	1.61 (0.34–3.16)	…
ART	14.63 (12.05–16.35)	1.60 (0.31–3.12)	…
Current INSTI-based ART	90 (32.4)	73 (83.9)	…

Data are presented as median (IQR) or No. (%).

Abbreviations: ART, Antiretroviral Therapy; HDL, high-density lipoprotein; HOMA-IR, homeostatic model assessment–insulin resistance; INSTI, integrase strand transfer inhibitor; LDL, low-density lipoprotein; YNPHIV, youth with nonperinatally acquired HIV; YPHIV, youth with perinatally acquired HIV.

In unadjusted analyses, there were overall differences in LGE mass (1.85 g among YPHIV, 2.00 g among YNPHIV, and 1.41 g among youth with HIV seronegativity; *P* < .001) and volume fraction (5.8%, 6.4%, and 4.5%, respectively; *P* < .001; Table 2). In addition, a greater proportion of YPHIV and YNPHIV exhibited the presence of LGE (67.6% and 72.6% vs 41.1%; overall *P* < .001). Mean native T1 time was highest in YNPHIV as compared with YPHIV and youth who were HIV seronegative (1221 vs 1208 and 1204 milliseconds; overall *P* < .001). T2 times at all sites were not significantly different among groups, nor were all myocardial strain indices.

**Table 2.** Cardiovascular Magnetic Resonance Imaging Measures of Subclinical Fibrosis, Inflammation, and Function by HIV Status

	YPHIV (n = 278)	YNPHIV (n = 87)	Youth Without HIV (n = 99)	*P* Value^a^	*P* Value^b^	*P* Value^c^
Subclinical myocardial fibrosis						
LGE						
Mass, g	1.85 (1.31–2.64)	2.00 (1.31–2.46)	1.41 (1.05–2.00)	<.001	<.001	<.001
Volume fraction, %	5.8 (4.7–7.2)	6.4 (4.9–7.9)	4.5 (3.7–5.6)	<.001	<.001	<.001
Presence of LGE, No. (%)	169 (67.6)	61 (72.6)	39 (41.1)	<.001	<.001	<.001
ECV, %						
Overall mean	27 (25–29)	28 (26–30)	27 (25–29)	.078	.14	.020
LV base	27 (25–29)	27 (26–29)	26 (25–28)	.029	.089	.008
LV mid	27 (25–29)	28 (26–29)	27 (24–28)	.070	.14	.026
LV apex	28 (26–31)	28 (27–30)	28 (25–29)	.030	.053	.007
Myocardial inflammation, ms						
Native T1 LV						
Mean	1208 (1183–1234)	1221 (1204–1253)	1204 (1183–1222)	<.001	.22	<.001
Base	1206 (1183–1230)	1222 (1204–1251)	1203 (1188–1219)	<.001	.58	<.001
Mid	1203 (1178–1234)	1215 (1199–1247)	1201 (1178–1220)	<.001	.32	<.001
Apex	1211 (1187–1240)	1222 (1204–1256)	1203 (1185–1220)	<.001	.057	<.001
T2 LV						
Base	38.3 (37.1–39.5)	38.7 (37.0–40.0)	38.4 (37.2–39.5)	.600	.82	.46
Mid	38.3 (37.2–39.5)	38.8 (37.5–40.2)	38.4 (37.3–39.6)	.217	.62	.27
Apex	38.7 (37.6–40.1)	39.1 (37.9–40.5)	38.8 (37.8–39.9)	.339	.96	.18
Myocardial function: strain indices, %						
Radial	34.4 (31.1–38.1)	34.1 (30.0–37.5)	33.8 (30–37.7)	.607	.41	.95
Midcircumferential	−19.8 (2.1)	−19.6 (1.8)	−19.6 (1.7)	.674	.43	.87
Longitudinal	−17.3 (2.1)	−17.3 (2.1)	−17.5 (2.0)	.666	.38	.48

Data are presented as median (IQR) or mean (SD) unless noted otherwise.

Abbreviations: ECV, extracellular volume; LGE, late gadolinium enhancement; LV, left ventricle; YNPHIV, youth with nonperinatally acquired HIV; YPHIV, youth with perinatally acquired HIV.

^a^Kruskal-Wallis test for 3-group comparison.

^b^Wilcoxon rank sum test for 2-group comparison of YPHIV vs HIV seronegative.

^c^Wilcoxon rank sum test for 2-group comparison of YNPHIV vs HIV seronegative.

**Figure 2.**
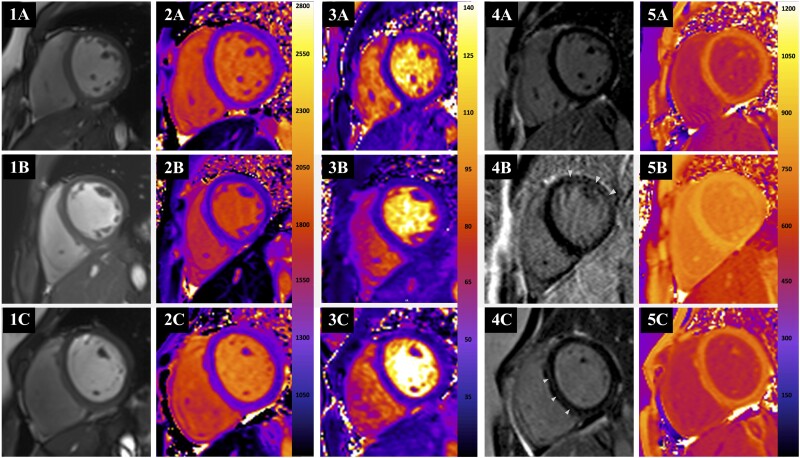
Representative examples of steady-state free precession images and corresponding precontrast T1 maps, T2 maps, late gadolinium enhancement images, and postcontrast T1 maps with their relevant values. Steady-state free precession image at end diastole: *1A*, youth who were HIV seronegative (YHIV–); *1B*, youth with perinatally acquired HIV (YPHIV); *1C*, youth with nonperinatally acquired HIV (YNPHIV). Precontrast T1 map: *2A*, YHIV– (1228 ms); *2B*, YPHIV (1171 ms); *2C*, YNPHIV (1245 ms). T2 map: *3A*, YHIV– (38 ms); *3B*, YPHIV (39 ms); *3C*, YNPHIV (40 ms). Late gadolinium enhancement image: *4A*, YHIV– with no enhancement; *4B*, YPHIV with anterolateral linear midwall enhancement (white arrows); *4C*, YNPHIV with septal linear midwall enhancement (white arrows). Postcontrast T1 map: *5A*, YHIV– (684 ms) with extracellular volume (ECV) of 0.26; *5B*, YPHIV (819 ms) with ECV of 0.25; *5C*, YNPHIV (696 ms) with ECV of 0.27.

After adjusting for age, female sex, body surface area, previous tuberculosis disease, and systolic blood pressure, median LGE mass (β = 0.468, *P* = .001, for YPHIV; β = 0.544, *P* = .002, for YNPHIV) and LGE fraction (percentage; β = 1.587, *P* < .001, for YPHIV; β = 1.781, *P* < .001, for YNPHIV) were higher in YPHIV and YNPHIV when compared with youth who were HIV seronegative; native T1 and ECV did not differ among groups in adjusted analyses (Table 3). Among youth with HIV, CMR outcomes did not differ significantly for persons with perinatal vs nonperinatal HIV after additionally adjusting for age at ART initiation, HIV RNA level, and ART based on integrase strand transfer inhibitor. Figure panels 1A, 2A, 3A, 4A, and 5A show representative precontrast T1 and T2 mapping, postcontrast T1 mapping, LGE, and ECV from CMR in youth who were HIV seronegative, while panels 1B, 2B, 3B, 4B, and 5B and 1C, 2C, 3C, 4C, and 5C represent the same in YPHIV and YNPHIV, respectively. Specifically, arrows in Figure 2 panels 4B and 4C demonstrate linear midwall LGE in individuals from the YPHIV and YNPHIV groups, as compared with an absence of LGE in a participant who was HIV seronegative (panel 4A). Findings generally did not vary in analyses restricted to females (Supplementary Table).

**Table 3.** Adjusted Mean Differences in CMR Measures by HIV Status

	Model Outcome							
	LGE Mass		LGE Fraction (Percentage)		ECV Mean		T1 Mean	
Exposure of Interest	Coefficient	*P* Value	Coefficient	*P* Value	Coefficient	*P* Value	Coefficient	*P* Value
Model (entire cohort)^a^								
YPHIV	0.468	.001	1.587	<.001	−0.001	.765	2.437	.676
YNPHIV	0.544	.002	1.781	<.001	0.002	.776	11.477	.102
HIV seronegative	Ref	…	Ref	…	Ref	…	Ref	…
Model (youth with HIV)^b^								
YPHIV	−0.153	.646	−1.183	.174	0.009	.269	6.482	.602
YNPHIV	Ref	…	Ref	…	Ref	…	Ref	…

Abbreviations: ART, antiretroviral treatment; CMR, cardiovascular magnetic resonance; ECV, extracellular volume; Ref, reference; INSTI, integrase strand transfer inhibitor; LGE, late gadolinium enhancement; YNPHIV, youth with nonperinatally acquired HIV; YPHIV, youth with perinatally acquired HIV.

^a^Adjusted for age, home running tap water, female sex, body surface area, history of tuberculosis, and systolic blood pressure.

^b^Additionally adjusted for age at ART initiation, HIV RNA level, and INSTI-based ART, as well as all covariates from the model including the entire cohort.

## DISCUSSION

In the largest study to date of CMR among youth with HIV in sub-Saharan Africa without significant CVD risk factors or comorbidity and with well-established ART, we found that despite their young age, YPHIV and YNPHIV appear to have higher subclinical myocardial fibrosis than youth with HIV seronegativity and healthy adults in South Africa. However, myocardial inflammation and function were not significantly different by HIV status.

To our knowledge, no other studies have been published assessing subclinical myocardial fibrosis via CMR in youth with HIV. In our previous smaller study of CMR in South African youth, we reported that relationships between left ventricle remodeling and myocardial contractility in YPHIV were opposite those seen in youth with HIV seronegativity [33]. Among YPHIV, increasing left ventricle mass/volume ratio (as a measure of left ventricle remodeling) was not associated with an expected improved global circumferential strain, as observed in youth who were HIV seronegative. This led to the hypothesis that YPHIV have subclinical myocardial fibrosis when compared with youth with HIV seronegativity. Our current larger study clearly demonstrates higher LGE mass in YPHIV and YNPHIV as compared with youth with HIV-seronegative status, though only YPHIV had a significantly higher LGE volume fraction when compared with youth who were HIV seronegative.

Our findings are consistent with adult studies reporting that PWH have increased myocardial fibrosis as compared with healthy adults who are HIV seronegative [6–8, 11, 13, 34]. We and others have shown that among adults without CVD, a significantly greater proportion of adult PWH exhibit the presence of LGE when compared with healthy adults with HIV seronegativity in South Africa [6, 34]. The majority of PWH in these studies were undergoing ART. Similar findings have been reported in PWH with ART in Europe and Peru [7, 9, 11]. Other adult studies on myocardial fibrosis in HIV reported higher ECV in PWH as compared with adults with HIV seronegativity [13, 17, 35]. We did not observe this in final adjusted analyses in our study, which may indicate that youth vs adults with HIV exhibit a more focal rather than diffuse nature of fibrosis, as LGE more broadly represents focal and ECV diffuse myocardial fibrosis.

Myocardial scar and fibrosis, as identified by the presence of LGE on CMR, are independent predictors of all-cause mortality, cardiovascular mortality, and heart failure hospitalization in adults with nonischemic heart disease, as well as in those with idiopathic cardiomyopathy [36]. Furthermore, the presence of LGE has been associated with subsequent CVD events, including death, myocardial infarction, and CVD-related hospitalizations [37]. The higher LGE mass that we observed in YPHIV and YNPHIV as compared with youth who were HIV seronegative, in the absence of discernible myocardial edema, is concerning and warrants further long-term follow-up.

HIV inflammation has been implicated as a mechanism through which the virus may contribute to myocardial fibrosis [10]. Several studies in adult PWH have shown higher native T1 [6, 7, 9, 11, 13] and native T2 [6, 9, 11, 13] vs adults with HIV-seronegative status. Native T1 mapping measures changes in the myocardial extracellular matrix, which can be associated with chronic inflammation, as well as collagen deposition and iron overload. Native T2 mapping, however, is a measure of acute and chronic inflammation. Native T2 was similar among the groups in our study, reflecting few differences by HIV status in inflammation among youth. While we did not observe differences by HIV status in native T1 in adjusted analyses, there were native T1 differences among the groups in unadjusted analyses, largely driven by higher native T1 in YNPHIV. In our study, YPHIV and YNPHIV had higher rates of prior tuberculosis disease, which may contribute to higher burden of myocardial T1 values. There are few published data on reference T1 and T2 mapping in healthy youth. However, one study of 119 youth in Spain, which utilized CMR techniques and methodology similar to ours, reported mean native T1 values of 1234 milliseconds, with girls having significantly higher values than boys [31].

Despite higher subclinical myocardial fibrosis in youth with HIV, myocardial function on CMR was similar among the groups, which is reassuring. This is in contrast to older studies assessing myocardial function by HIV status in children via echocardiography but consistent with later echocardiography studies demonstrating that YPHIV with ART have better myocardial function than YPHIV born in the pre-ART era [21]. All youth with HIV in our study were undergoing ART, with the majority of YPHIV having initiated ART in the first few years of life and with the majority of YNPHIV having been receiving ART for more than a year prior to enrollment, which may partially explain the lack of differences in myocardial function by HIV status in our study. Last, we did not find differences in any CMR outcome measures by mode of HIV acquisition. Smaller sample sizes should be taken into account when interpreting this, but there is the possibility that the YPHIV in our study, having initiated ART early in life, are not as dissimilar from their YNPHIV peers in overall myocardial function during adolescence.

Inferences from this study were limited by its cross-sectional study design and location in 1 high-burden setting in South Africa. However, this country has one of the highest number of adolescents with HIV globally. In our study, females represented a larger proportion of YNPHIV than YPHIV or youth who were HIV seronegative, likely reflecting the real-world HIV epidemiology in South Africa, where the incidence of HIV among individuals 15 to 24 years of age is 5 times higher in females than males [38]. It may be that the finding that we observed in our overall models simply reflects differences among YPHIV, YNPHIV, and youth with HIV seronegativity that are specific to females. However, we adjusted for female sex, and in sensitivity analyses restricted to females, we observed similar findings as our primary models including the entire cohort. Due to insufficient sample sizes of males in all groups, we were unable to test for sex differences in the relationship between HIV status and CMR outcomes. There is also the potential for unmeasured confounders, particularly since there were differences among the comparison groups in the covariates within our models as well as in social determinants of health, which were not fully collected. Last, we did not account for multiple testing, although if we had considered α < .006 as significant (accounting for 4 related outcomes, which were the outcomes that we modeled, and 2 comparisons each), our overall findings of higher LGE mass and fraction in YPHIV and YNPHIV vs youth who were HIV seronegative would still be considered significant.

In conclusion, YPHIV and YNPHIV with ART and without significant CV comorbidity appear to have higher subclinical myocardial fibrosis than youth with HIV seronegativity and healthy adults in South Africa. As CVD and HIV are both significant public health burdens in sub-Saharan Africa, this important population may benefit from early screening for CVD and timely initiation of cardioprotective therapies to prevent development of heart failure.

## Supplementary Data

Supplementary materials are available at *Open Forum Infectious Diseases* online. Consisting of data provided by the authors to benefit the reader, the posted materials are not copyedited and are the sole responsibility of the authors, so questions or comments should be addressed to the corresponding author.

## Supplementary Material

ofae555_Supplementary_Data

## Notes

***Acknowledgments.*** We thank all the study participants and staff at the University of Cape Town and Red Cross War Memorial Children's Hospital in Cape Town, without whom this study would not have been possible.

***Author contributions*.** J. J., H. J. Z., and N. A. B. N. conceptualized the study. J. J. wrote the first draft of the manuscript and took primary responsibility for coordinating revisions and finalizing the manuscript. N. A. A.-A., J. L., N. J., E. C., and T. M. collected and cleaned data. M. K., S. J., D. E., and N. A. B. N. made significant contributions to the interpretation of CMR data and writing of the CMR method description. N. A. A.-A., E. C., L. M., and L. B. B. gave input on the methods. M. N. N. and L. M. analyzed the data, and L. B. B. helped edit final versions of the Methods section. G. A. M., M. F., I. J. K., L. M., and H. J. Z. gave overall input on the structure of the article with focus on the Discussion. N. A. B. N. gave substantial input on the discussion. All authors reviewed the manuscript and approved the final version.

***Data availability.*** Data are not publicly available but may be made available upon request if needed.

***Previous presentation.*** These data were presented in part at the European Society of Cardiology Congress, August 2023, Amsterdam, the Netherlands.

***Financial support.*** This work was supported by the National Heart, Lung, and Blood Institute (NHLBI) (R01HL151287); Eunice Kennedy Shriver National Institute of Child Health and Human Development (R01HD074051); and the South African Medical Research Council (to H. J. Z.).
