# Supplementary material for: Subclinical Myocardial Fibrosis in South African Youth With HIV: Results From the CTAAC-Heart Study
Source: Open Forum Infect Dis. 2024 Oct 1;11(10):ofae555. doi: 10.1093/ofid/ofae555 (PMC11482013; doi:10.1093/ofid/ofae555)
Supplement: ofae555_Supplementary_Data [file ofae555_supplementary_data.docx]

**Supplemental Table. Adjusted mean differences in CMR measures by HIV status among females**

|  | **Model Outcome** | | | | | | | |
| --- | --- | --- | --- | --- | --- | --- | --- | --- |
|  | **LGE Mass** | | **LGE Percentage** | | **ECV mean** | | **T1 Mean** | |
| **Exposure of Interest** | **Coefficient** | ***p* value** | **Coefficient** | ***p* value** | **Coefficient** | ***p* value** | **Coefficient** | ***p* value** |
| ***Model (Entire cohort)*^1^** | | |  |  |  |  |  |  |
| YPHIV | 0.442 | *0.003* | 1.480 | *0.003* | -0.002 | *0.778* | 5.940 | *0.496* |
| YNPHIV | 0.504 | *0.001* | 1.675 | *0.002* | 0.001 | *0.848* | 11.973 | *0.206* |
| HIV seronegative | Ref | *--* | Ref | -- | Ref | -- | Ref | -- |
|  |  |  |  |  |  |  |  |  |
| ***Model (Youth with HIV)*^2^** | | |  |  |  |  |  |  |
| YPHIV | -0.234 | *0.518* | -0.271 | *0.813* | -0.014 | *0.262* | -16.128 | *0.400* |
| YNPHIV | Ref | -- | Ref | -- | Ref | -- | Ref | -- |
|  |  |  |  |  |  |  |  |  |
| *^1^ Adjusted for age, home running tap water, body surface area, history of previous tuberculosis, systolic blood pressure*  *^2^ Additionally adjusted for age at ART initiation, HIV RNA level, and INSTI-based ART as well as all covariates from the model including the entire cohort*  *ART=antiretroviral treatment, ECV=extracellular volume, INSTI=integrase strand transfer inhibitor, YNPHIV=youth with non-perinatally acquired HIV, YPHIV=youth with perinatally acquired HIV* | | | | | | | | |
